# Selective Scandium Elution from D_2_EHPA-Impregnated Ion-Exchange Resin After Metal Loading from Acidic Chloride Solutions

**DOI:** 10.3390/ma17246089

**Published:** 2024-12-13

**Authors:** Eleni Mikeli, Danai Marinos, Efthymios Balomenos, Dimitrios Panias

**Affiliations:** Laboratory of Metallurgy, School of Mining and Metallurgical Engineering, National Technical University of Athens, Iroon Polytechniou 9 Str., Zografou Campus, 15773 Athens, Greecethymis@metal.ntua.gr (E.B.); panias@metal.ntua.gr (D.P.)

**Keywords:** scandium extraction, ion exchange, D2EHPA-impregnated resin and elution

## Abstract

This paper investigates the elution behavior of scandium from D_2_EHPA (Di-(2-ethylhexyl) phosphoric acid)-impregnated resins that proceed with metal loading from acidic chloride solutions. D_2_EHPA resins stem from their recognized selectivity for Sc extraction from acidic solutions. This study focuses on the elution process after ion-exchange extraction and examines various elution systems to achieve selective Sc recovery. Among the tested elution media, fluoride-based systems were proven effective for Sc desorption. The elution of the resins was demonstrated in a column set-up, where complete and selective elution of Sc was achieved. Τhis study contributes to the advancement of Sc extraction methods from chloride solutions, offering valuable insights for industrial applications, especially emphasizing the importance of optimizing the elution step for achieving efficient recovery of Sc.

## 1. Introduction

Scandium (Sc), due to its unique properties, holds significant promise for industrial applications for advancing environmental sustainability and decarbonization. It can be applied in a wide range of industries, including solid oxide fuel cells (SOFCs) as a high-efficiency electrolyte, lightweight alloys for aerospace and automotive industries, and metal halide lamps for improved lighting efficiency [1,2]. Scandium does not have a strong attraction to common ore-forming anions, leading to its widespread distribution in the lithosphere and its occurrence in low concentrations within more than 100 different minerals [3]. As a result, the primary production of Sc is limited, and it is typically produced as a secondary material from other metallurgical industries including iron, rare earths, titanium, and zirconium in China, uranium in Kazakhstan, and nickel in the Philippines [3]. The annual production of Sc is estimated to be between 14 and 23 tons, but there are estimates that the production, potentially, might exceed 1800 tons at its peak [2]. China holds the maximum share of scandium supply, accounting for 67% of the market. Despite its critical importance, there is currently no scandium production in the EU and it is listed as a critical raw material due to its high supply risk and economic importance [4].

However, the titanium industry generates scandium-rich by-products, offering significant potential for scandium production within the EU [5]. The “chloride process” creates about 0.5 million tons of TiO_2_ pigment per year in Europe. This technique leaves iron-rich residual solutions with exploitable sources of scandium (60–140 mg/L) [6]. These solutions are either neutralized and landfilled at a significant cost or sold as a by-product without valorization of their Sc content. Purifying scandium streams is particularly challenging because the impurities typically present are found at concentrations more than ten times higher than scandium. Solvent extraction (SX) is the most employed and commercially acceptable method for Sc extraction from acidic solutions [1]. There is a wide range of extractants that have been applied to extract and separate Sc from acidic solutions. Generally, organophosphorus compounds are found to be efficient for selective Sc extraction from TiO_2_ acid waste streams. In particular, bis (2-ethylhexyl)-phosphoric acid, (also referred to as D_2_EHPA, HDEHP, or P_2_O_4_), tri-n-butyl-phosphate (TBP), or synergistic systems of these extractants are the most widely used and commercially acceptable reagents for Sc extraction [6].

Recently, ion-exchange (IX) processes have gained a lot of attention for Sc extraction, as they offer several advantages for SX, including reduced contamination of the pregnant solution, less complicated flow sheets, less extractant loss, avoidance of emulsion, and lower operating costs [7,8,9]. Ion-exchange procedures for Sc extraction typically utilize polymers that are functionalized with phosphate functional groups or impregnated with organophosphorus solvents, where the extraction mechanisms of metals are expected to be similar to SX procedures. Mostajeran et al. studied a series of ion-exchange resins for the recovery of Sc from synthetic and actual pregnant leach solutions obtained from the acid leaching of coal fly ash [10]. Among the tested resins, Sc was adsorbed quickly on TP 272 and Lewatit VPOC 1026, with the latter showing the highest capacity for Sc. Toli et al. studied a series of commercial ion-exchange resins for the recovery of Sc from actual pregnant leach solutions obtained from the acid leaching of bauxite residue. It was found that Seplite LSC790, an aminomethyl phosphonic resin and a D_2_EHPA-impregnated resin, had the most significant capacity for Sc while maintaining high selectivity towards Ti co-extraction [11]. In our previous work, the performance of two different phosphorous-based IX resins was studied for their ability to extract Sc from high-concentration aqueous solutions derived from the TiO_2_ industry. It was concluded that the D_2_EHPA-impregnated resin, Lewatit VPOC1026, shows great potential to extract Sc selectively from such solutions [12].

D_2_EHPA is a weak organophosphorus acid, and its main characteristic is the formation of a hydrogen bond between its molecules, which results in the formation of dimeric structures [13]. The solvent extraction mechanism for metals such as scandium has been suggested to be a cation-exchange reaction, where Sc forms a complex with the dimeric D_2_EHPA molecules, why is expressed in Equation (1) [1], in low acidity systems. However in high acidity systems, it has been suggested that solvation extraction may also take place, which is expressed by in Equation (2) [13]. For the IX extraction using D_2_EHPA-impregnated resins, the metals’ extraction is expected to show similar extraction mechanisms to the SX procedure. However, due to the high specific surface of such resins, other sorption mechanisms might also take place during metal extraction with IX. For Sc, the only stable oxidation state is Sc (III) in aqueous solutions, and, when it comes to acidic chloride solutions (pH ≤ 4), the predominant speciation is *Sc*^+3^ [14].

(1) $${Sc}_{\left(aq\right)}^{+3}+3{\left(RH\right)}_{2(org)}⇌Sc{{(R}_{2}H)}_{3(org)}+{3H}_{\left(aq\right)}^{+}$$

(2) $${Sc}_{\left(aq\right)}^{+3}+{3Cl}_{\left(aq\right)}^{-}+n{\left(RH\right)}_{2(org)}⇌Sc{Cl}_{3}{\;*\;n(RH)}_{2(org)},\;n\geq 1/2$$

The desorption process (i.e., elution or stripping) of the loaded resins is the opposite procedure of loading, with its reaction following the opposite direction of Equations (1) and (2). During desorption, the loaded metals are selectively recovered from the resin in increased concentrations into an aqueous stream for downstream purification.

The desorption process is an essential part of the metal recovery in IX procedures, but its importance is usually neglected. Generally, scandium-hydrated ions, categorized as Pearson hard acids due to their high oxidation state, tend to form stable complexes with hard ligands such as hydroxide, fluoride, sulfate, and phosphate [14]. As such, ligands containing these groups are potential candidates for elution agents. For solvent extraction (SX) applications involving D_2_EHPA, alkaline solutions like NaOH or Na_2_CO_3_ are commonly employed to elute scandium as stable hydroxide or carbonate complexes [15]. Efficient scandium (Sc) elution from phosphorus-containing ion-exchange resins has been reported in some studies, utilizing agents such as Na_2_CO_3_ and fluoride salts (e.g., NaF, NH_4_F) [10,16,17]. However, for D_2_EHPA-impregnated resins, the manufacturer recommends operation at pH > 4, limiting the use of alkaline or neutral elution agents. Additionally, scandium ions, with their small ionic radius and high charge, exhibit a strong affinity for forming stable organic complexes [18]. This has led to the testing of miscible organic solutions, including oxalic acid and mineral acid mixtures containing organic additives such as EDTA, NTA, or alcohols, as elution agents in some studies [19,20]. Mineral acids are not efficient for Sc desorption. Mostajeran was unable to efficiently remove Sc from a loaded D_2_EHPA-impregnated resin using 2 M and 5 M H_2_SO_4_ solution, while in a more recent study, a mixture of 4 M H_2_SO_4_ and 40% vol. propanol eluted 98% of the loaded Sc [10,21,22].

While significant progress has been made in highlighting the great potential of D_2_EHPA-impregnated resins to selectively recover Sc from leachates, the elution procedure remains a big challenge. This study addresses this gap by investigating selective and efficient elution methods, focusing on fluoride-based systems and impurity management techniques to optimize scandium recovery in a complete IX cycle. In particular, a proper desorption process of the Sc-loaded D_2_EHPA resins was investigated. Initially, batch elution experiments were first conducted using various reagents to evaluate their effectiveness. The focus then shifted to hydrofluoric acid (HF), with batch experiments performed at different HF concentrations. Subsequently, column experiments were carried out, using low-concentration HF for impurity removal, including a recycling procedure of the eluate within the column. Finally, high-concentration HF was applied to recover scandium effectively. From the obtained results, the potential application of D_2_EHPA-impregnated resins on a larger scale was discussed. The goal of this study is to provides valuable insights into enhancing the efficiency and selectivity of Sc elution from D2EHPA impregnated resins processes, that could be applied for Sc extraction from various hydrochloric acid leachates, including ilmenite leachates, bauxite residue leachates and slag leachates.

## 2. Materials and Methods

### 2.1. Raw Materials and Reagents

For the investigation of Sc desorption from D_2_EHPA-impregnated resins, two different D_2_EHPA-impregnated ion-exchange resins were used: (1) Lewatit VPOC1026 provided by LANXESS (Cologne, Germany) and (2) Seplite LSC790 provided by SUNRESIN (Xi’an, China). For the elution tests, resins were preloaded either with a synthetic solution containing Sc or with an FeCl_2_ solution that was obtained through actual titanium oxide production industry. The loading of the resins was based on the optimum loading conditions demonstrated in our previous work [12]. Wet chemical analysis of the samples was carried out using the Inductively Coupled Plasma–Optical Emission Spectrometry (ICP OES), Optima 8000 by Perlin Elmer (Waltham, MA, USA). Calibration standard solutions were prepared from commercially available ICP, Ti, Sc, V, Zr, and Fe standards (1000 ppm) obtained from Merck (Darmstadt, Germany).

### 2.2. Fixed-Bed Column Loading and Elution Experiments

The loading and elution procedures of the D_2_EHPA-impregnated resins were demonstrated in fixed-bed column set-up utilizing laboratory polypropylene columns with 7 mL bed volume. The top and bottom of the column had fixed caps with Luer-lock inlet and outlet fittings for tubing attachment. D_2_EHPA resin of known weight was packed into the column. The feed solution continuously passed through the column in an upward constant flow of 1 BVh^−1^, using a peristaltic pump LabDos from Hitec Zang (Herzogenrath, Germany). On the top of the column, fractional samples of the effluent solution were collected for chemical analysis of the metals of interest.

For some experiments, a fixed amount of eluate solution was recycled in a closed loop into the column. Samples were taken at different times to measure the metal concentration.

### 2.3. Batch Elution Experiments

For the elution experiments, performed in batch set-up, an amount of loaded resin of known weight was added to glass Erlenmeyer flasks with a known volume of eluate solution. The flasks were plugged with a glass stopper and shaken in an incubator shaker at a fixed rotation speed of 220 rpm, ambient temperature, and retention time of 24 h. After completion of the batch experiment, the resin and solution were separated through vacuum filtration and the solution was prepared for chemical analysis. The elution efficiency was calculated by Equation (3).

(3) $$\mathrm{Elution}\;\mathrm{Efficiency}\;(\%):\;\%E=100\%:\;\frac{C_{f}\cdot \;V_{f}}{m_{i}}$$

where $C_{f}$ is the concentration (mg/L) of metal in the eluent solution, $V_{f}$ is the eluent volume (L), and $m_{i}$ is the mass (mg) of loaded metal on the resin used in the experiment.

## 3. Results and Discussion

### 3.1. Desorption of Metals from D_2_EHPA-Impregnated Resins

In the case of extracting elements that exist in very low concentrations compared to the corresponding impurities, it is essential to utilize selective reagents. Besides their selectivity, the selected agents should be easily eluted to efficiently back extract the valuable metals in clean streams for further processing and purification. The selectivity of the reagents to specific metals is related to the strong affinity between the reagent and the target metal, which could pose an important challenge in the elution stage. Even though there is significant research on selective IX extracting agents, their elution behavior is usually neglected. In this section, an investigation of possible effective eluents is conducted.

#### 3.1.1. Batch Elution Screening Tests

The common practice for elution (i.e., stripping) in solvent extraction applications, where D_2_EHPA is widely used for scandium extraction, is the use of alkaline solutions like sodium hydroxide or sodium carbonate to elute scandium in the form of the stable hydroxide and carbonate complexes accordingly [15]. These elution systems are not recommended in the case of D_2_EHPA-impregnated resins, since the stability of the resins’ active material is compromised at a pH above 4 [23,24], so acidic elution systems should be applied.

**Figure 1:**
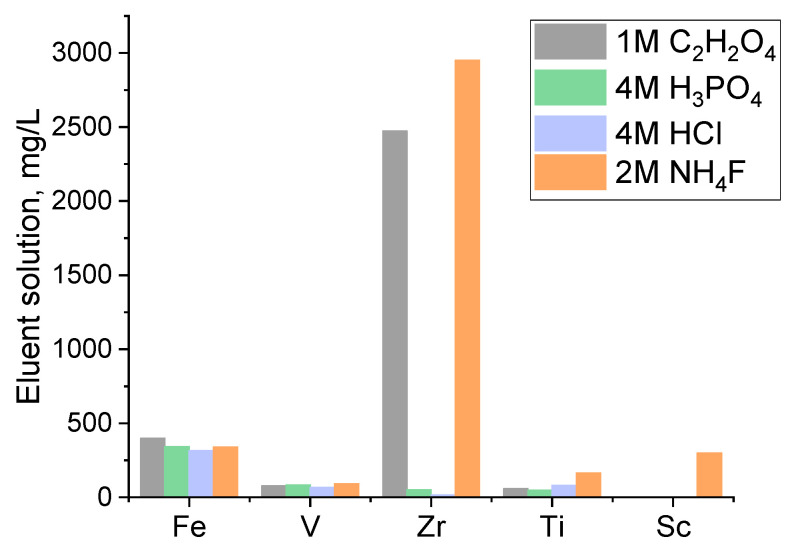
Concentration of the eluate solution after batch elution experiment of the metal loaded D_2_EHPA resin with various agents, resin/solution ratio 1/10, ambient temperature, 24 h.

Initial screening elution experiments were performed using selected reagents: phosphoric acid, hydrochloric acid, oxalic acid, and ammonium fluoride salt as elution ligands. The anions Cl^−^, F^−^, and C_2_O_4_^−2^ are common ligands known to form complexes with metals, with the increasing ligand field strength as follows: Cl^−^ < F^−^ < C_2_O_4_^−2^ [25]. Moreover, the phosphoric acid had been successfully tested in previous studies [26]. The resin tested in these experiments was priorly loaded with the actual FeCl_2_ solution as described in our previous work [12]. The results, illustrated in Figure 1, indicate that the choice of eluent did not have an impact on the elution of Fe and V, as a similar concentration was achieved for these metals in the eluent solution regardless of the type of the eluent tested. This behavior could indicate the low affinity these elements have with the resin, which results in an easy elution. For Ti, a considerably increased concentration was observed with the use of NH_4_F compared to the other eluents, while the concentration of Zr, and Sc was significantly higher compared to the other eluents.

Especially for Sc elution, NH_4_F was the only eluant that was efficient, showcasing that among the tested eluants, only the fluoride one could effectively be used for Sc elution. Consequently, the batch elution experiments show that fluoride salt is the most effective elution agent for every metal, as it produces eluent solutions with the highest metal concentrations for all the measured elements.

This effectiveness for Sc elution with the fluoride salt can be attributed to the strong affinity of scandium-hydrated ions, categorized as Pearson hard acids, for fluoride ligands, which are considered hard bases [14]. The fluoride ions form highly stable scandium–fluoride complexes (e.g., ScF^2+^, ScF_3_, ScF^4−^, ScF_6_^3−^) due to the small ionic radius and high charge density of scandium ions, which enhance the interaction between the metal and ligand [18]. This observation is consistent with previous studies demonstrating the efficiency of fluoride salts like NaF and NH_4_F in eluting scandium from phosphorus-containing resins [10,17,26,27,28]. Mosterajan et al., who also tested various reagents for the elution of Sc from loaded D_2_EHPA resins, showed that Sc elution from the D_2_EHPA resin with mineral acids and various salts was negligible while the alkaline solutions led to the formation of precipitates. Among the agents tested, the elution of Sc was achieved in a 2 M NH_4_F solution [10]. It should be noted though that aqueous solutions of fluoride salts have a pH > 4, meaning they could compromise the resins’ structure based on the manufacturer’s datasheet [23,24].

Interesting elution behavior was also observed with oxalate-based ligand, which has the highest ligand strength among the eluents tested [25] and has been effectively used for the extraction of Sc from aqueous solutions [29,30] since they are known to form strong oxalate complexes Sc(ox)_3_^−3^ [31]. The results in Figure 1 show that the use of oxalic acid as an eluent results in an eluent with a considerably high concentration of Zr with no Sc. This property could be utilized as a scrubbing process for the selective removal of Zr, which is a major impurity, leading to more selective Sc elution. However, it has been reported that elution with oxalic acid can lead to solid precipitate formation on the surface of the resin [19]. In such cases, the resin surface is partially blocked, which complicates the overall resin regeneration by requiring an additional step for precipitate dissolution.

For further screening elution experiments, a D2EHPA-impregnated resin previously loaded with a synthetic solution containing only Sc was used. Different fluoride-based systems (NH_4_F/HCl, KF/HCl, HF) were tested in acidified conditions, ensuring the resin’s structure stability by maintaining a pH below 4. The potential use of some organic complexing agents (NTA, EDTA) and the use of inorganic acids (HCl and H_2_SO_4_) at ambient temperature and 50 °C were also investigated. The concentration of the elution systems tested was either equal to the stoichiometrically needed concentration or at a higher concentration, and the results are summarized in Table 1.

In the case of organic complexing agents (NTA and EDTA salts), the resulting elution solutions were not stable at the desired conditions due to their very low solubility. The results in Table 1 prove that the only eluants that achieved scandium extraction were the fluoride ones at high concentrations. It should also be noted that the acidification of fluoride salts is challenging due to the common ion effect, which is higher at higher fluoride concentrations.

#### 3.1.2. Batch Elution Tests with HF

**Figure 2:**
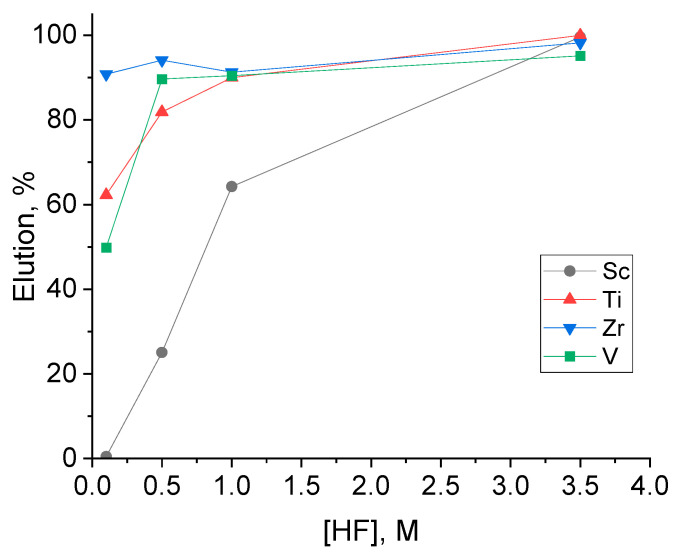
Batch elution experiments of loaded resin at various HF concentrations, resin/solution ratio 1/40, ambient temperature, 24 h.

From the above-mentioned observations, it was concluded that a fluoride system would be the most attractive option for Sc elution from D_2_EHPA resins. Aqueous solutions of fluoride salts (e.g., KF or NH_4_F) have a pH > 5, meaning they compromise the resins’ structure based on their datasheet [23,24]; thus, HF was selected as the elution agent for further investigation of the elution process. Batch experiments were performed with an HF solution of different concentrations (0.1–3.5 M) using resin that was previously loaded with FeCl_2_ solution, and these results are presented in Figure 2.

As observed in Figure 2, complete Sc elution is achieved only in high acid concentrations. Kaya et al. estimated that the increase in F^−^ concentration favors the formation of ScF_6_^3−^, which is highly soluble [26].

Moreover, it was also observed that a partial extraction of the metal impurities is achieved in low acid concentrations (0.1–0.5 M HF), where more than 80% of the metals are extracted even at 0.5 M HF. It is estimated that the easy elution of Zr, Ti, and V can be attributed to the weaker affinity of those metals to the resin. From the results, it can be deduced that the D_2_EHPA-impregnated resin forms less stable complexes with those metals compared to scandium in the initial loading step. Previous solvent extraction studies have shown that extraction systems with solely D_2_EHPA solvent show poor extraction behavior for Ti [32] while V can be stripped easily at low acid concentrations [33]. The weaker complexes between the resin and the impurities allow for their elution even at lower acid concentrations. In contrast, scandium, due to its smaller ionic radius and higher charge density, forms much stronger complexes with the resin, making it harder to elute. The resin has a higher affinity for scandium and, as a result, higher fluoride concentrations are required to break the more stable scandium–resin complexes and achieve complete elution. This explains why impurities are more easily extracted at lower HF concentrations while complete scandium extraction requires higher acid concentrations to overcome the stronger binding forces With this observation, the potential for applying a scrubbing step to remove the metal impurities is also worth considering.

**Figure 3:**
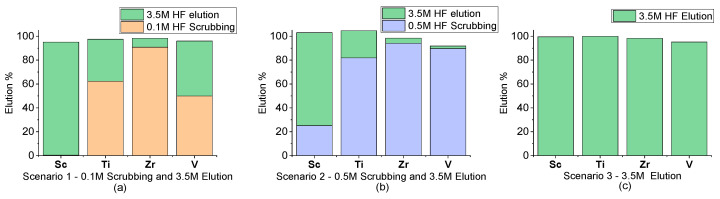
Batch elution experiments in three different elution scenarios, resin/solution ratio 1/40, ambient temperature, 24 h (**a**) scrubbing with 0.1 M HF, (**b**) scrubbing with 0.5 M HF (**c**) without scrubbing step.

In Figure 3, three different scenarios for the elution of the resin are presented and compared:

1. The first two scenarios involve a two-step elution process. In the first step, either 0.1 M HF or 0.5 M HF is used to scrub metal impurities. In the second step, 3.5 M HF is used to extract Sc.
2. The third scenario involves only a one-step elution with 3.5 M HF for complete metal extraction.

The results presented in Figure 3 show that complete metal removal can be achieved when applying the high-concentration HF. The two-step elution can achieve significant separation in the metal impurities with Sc and achieve selective Sc extraction in the second elution step. Table 2 presents the ratios of Sc towards metal impurities in the initial FeCl_2_ solution and the final Sc-rich solutions in the three proposed scenarios. Comparing the relevant concentration of Sc to the metal impurities in Table 2, it is shown that in all three elution scenarios, the final Sc concentration increased compared to the other metal impurities. Moreover, even though all the proposed scenarios recover 100% Sc from the loaded resin, Sc has a significantly increased concentration compared to the metal impurities when a scrubbing step is applied.

Even though the scrubbing step with 0.5 M HF achieves the best separation ratios for Sc compared to the other metals, more than 20% of the loaded Sc is lost during the scrubbing. Adjusting the acid concentration during the scrubbing step can determine the trade-off between the purity of the final Sc-rich solution and the loss of Sc recovery in the first step.

#### 3.1.3. Elution in Fixed Bed Columns

**Figure 4:**
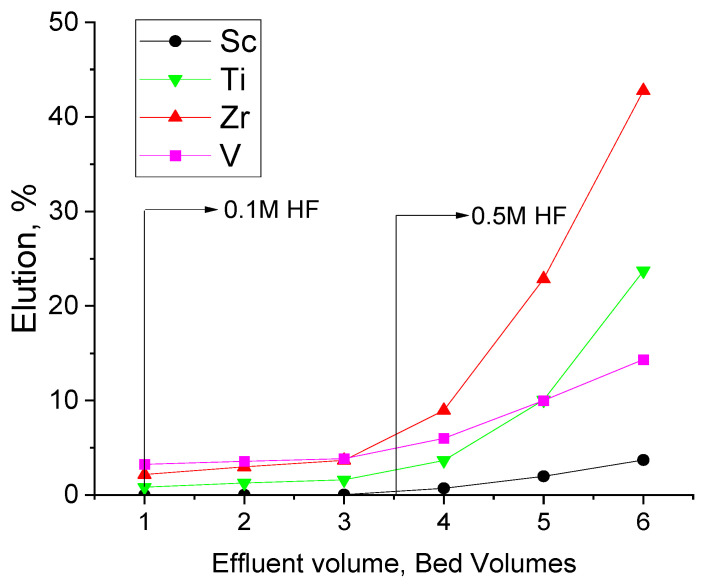
Cumulative elution of metals using 0.1 M HF and 0.5 M HF for 3 BV.

Based on the batch elution experiments, column experiments were carried out with 0.1 M HF in a column set-up for 3 bed volumes of effluent solution, where a very low cumulative elution rate (below 5%) was observed for the metals, as shown in Figure 4. This behavior contradicted the observations of the previous batch experiments concerning the metals, except Sc. In the batch experiments with 0.1 M HF, more than 50% extraction of Ti, Zr, and V was observed with no Sc co-extraction (Figure 3), and, in the same acid concentration, less than 5% of the metals were extracted in the column set-up experiments (Figure 4). To boost the elution performance in the column experiments, 3 additional bed volumes of 0.5 M HF passed through the same column, where the total elution rate still did not exceed 45% for Zr, 25% for Ti, and 12% for V, while Sc elution remained below 5%.

This observation, on the one hand, is explained by the fact that the amount of solution used in the batch experiments was proportionally larger and would be equivalent to 118 BV compared to the 3 BV that passed in the column set-up. It should be noted that in actual large-scale applications of IX, the minimum solution volume should be applied to achieve a high concentration of metals in the final solutions. On the other hand, batch experiments are also equilibrium experiments, which is not the case for column experiments. Column experiments are dynamic processes where the contact time of the resin with the solution is very short. Even though the chosen flowrate 1 BV/h is empirically considered a minimum flowrate in applications of ion-exchange process on a large scale, it was still not sufficient to achieve the elution rates observed in the batch experiments. Inferentially, the poor scrubbing performance in the column experiments was attributed to the very slow elution kinetics of the metals.

**Figure 5:**
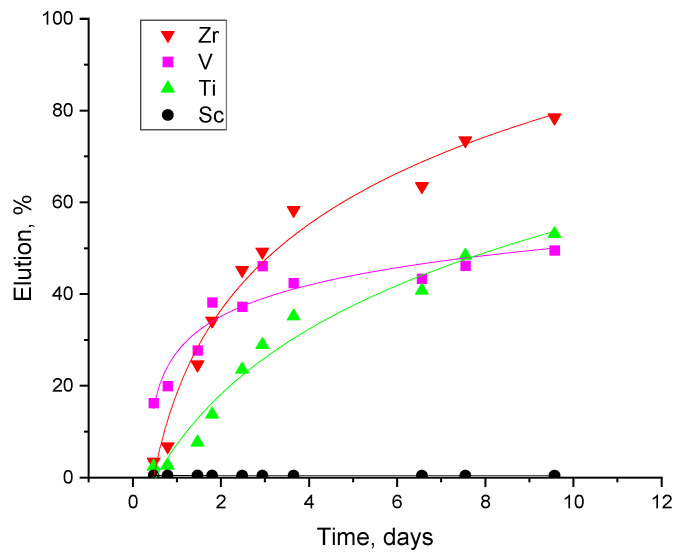
Kinetic elution experiment in column set-up recycling a fixed amount of 0.1 M HF solution in column set-up with 1 BV/h.

To verify the assumption that the poor kinetics of the column impend sufficient metal elution, a new experimental set-up was proposed, where the eluate solution was recycled in a closed loop in the experiment. For this experiment, the volume of the eluate solution used was 40 mL per g of resin to match the 1/40 resin/solution ratio that was used in the batch experiments (Figure 3). The results of this kinetic experiment in column set-up are presented in Figure 5.

The solution volume and concentration were set to replicate the exact conditions of the batch elution experiment (scrubbing with 0.1 M HF, ratio 1/40 g resin/mL solution, ambient temperature). Samples were taken at different times to measure the metal concentration. From the results presented in Figure 5, it is seen that the metal impurities needed over 9.5 days to reach a high concentration in the eluent solution, reflecting the very poor kinetics in the resin desorption. In the 9.5 days of the solution recycling, the elution rate of Zr, V, and Ti were calculated at 80%, 50%, and 53%, respectively, while Sc was not eluted. The elution rates in the column experiments are in agreement with our previous observations on the batch experiments and highlight the importance of kinetics in the resin’s performance.

**Figure 6:**
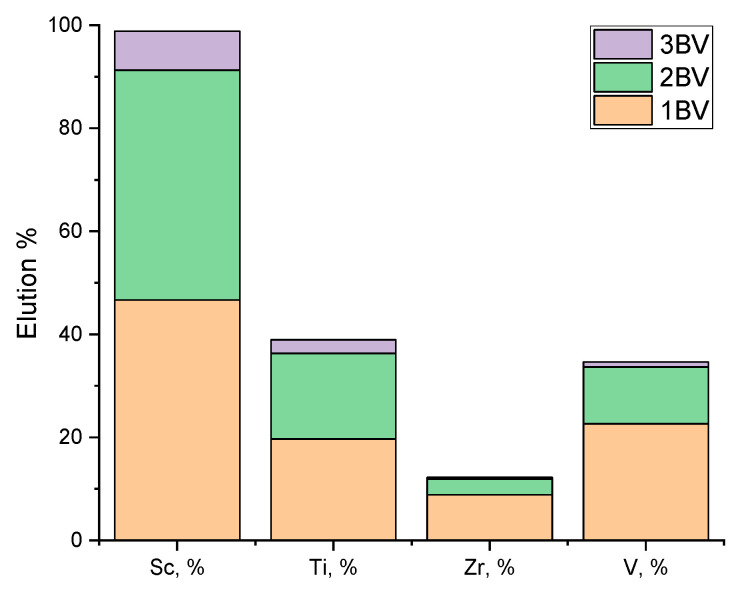
Second-step elution of resin with 3.5 M HF, for 3 BV solution, 1 BV/h^−1^.

After the scrubbing step with 0.1 M HF (with the recycling of the eluate), an elution step with 3.5 M HF was also conducted. For the second-step elution, 3.5 M HF was used and 3 BV of the solution passed through the column. The effluent solution was collected in three fractions of 1 BV each. It is shown in Figure 6 that most of the metals were removed at the first 2 BV and complete Sc extraction was achieved at 3 BV.

**Figure 7:**
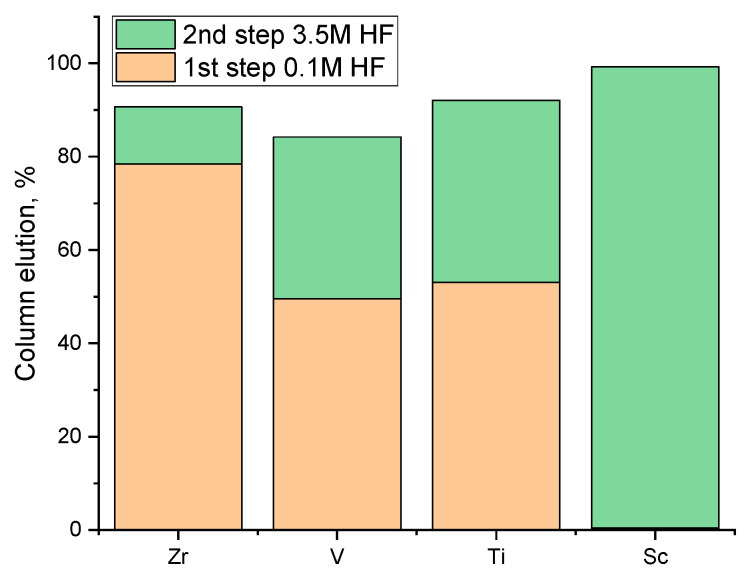
Elution rate distribution in the two-step elution process of the D_2_EHPA-impregnated resin using HF.

Figure 7 illustrates the summary of the two-step elution in a column set-up where most of the V, Zr, and Ti can be removed with an initial scrubbing step using a low concentration of HF (0.1 M). The metal impurities that remain loaded in the column after the 0.1 M HF scrubbing are also co-extracted in the second 3.5 M HF elution.

The feed solution and the final elution streams are presented in Table 3 for comparison.

## 4. Conclusions

In this study, the application of D_2_EHPA-impregnated resins for Sc IX extraction was investigated and the critical importance of thoroughly investigating the elution step was emphasized. Only high-concentration fluoride systems were found to be effective in recovering scandium from the D_2_EHPA-impregnated resins, highlighting the need for the optimization of the elution procedure. It is also highlighted in this study that impurity scrubbing techniques, such as using low-concentration HF, can selectively remove interfering metals before ion exchange. Additionally, it was observed that the kinetics of the metal extraction reactions are of great importance and are the key to fine-tuning the procedure to achieve the optimum balance of high extraction rates and high concentrations in the eluate solution.

In the two-step elution procedure applied, the initial application of 0.1 M HF effectively scrubbed metal impurities such as zirconium, vanadium, and titanium from the resin. This initial step achieved a significant reduction in impurity by removing Zr, V, and Ti with elution rates of 80%, 50%, and 53%, respectively, while Sc concentration was <2.5 ppm, indicating a minimal loss of scandium. The subsequent application of 3.5 M HF for scandium recovery yielded a concentrated elution with 305 ppm Sc, increased by 2.4 times compared to the initial feed solution along with substantially reduced impurity levels compared to the initial feed solution (23.6 times less Zr, 149.7 times less V, 5.95 times less Ti). This two-step approach demonstrated the effectiveness of selective scrubbing followed by targeted elution, resulting in an enhanced scandium concentration and minimized interference from other metals, thereby optimizing the elution process for Sc recovery.

Overall, the elution procedure described in this work is versatile and can be applied to a variety of hydrochloric acid leachates derived from various sources, such as ilmenite, bauxite residue, and slag leachates. Finally, further research is required on the treatment of the solution after the elution stage to ensure efficient downstream processing and improve the overall recovery rates, enhancing the sustainability of the overall scandium extraction processes.

## Figures and Tables

**Table 1:** Screening elution batch experiments performed in D_2_EHPA resin loaded with a synthetic Sc solution.

Elution System	Temperature, °C	Sc Elution %
0.1 M NH_4_F/0.1 M HCl	Ambient	<1.5
0.1 M NH_4_F/0.1 M HCl	Ambient	<1.5
0.1 M HF	Ambient	<1.5
0.1 M HF	Ambient	<1.5
0.1 M KF/0.1 M HCl	Ambient	<1.5
0.1 M KF/0.1 M HCl	Ambient	<1.5
3.5 M NH_4_F/1.3 M HCl	Ambient	100
3.5 M HF	Ambient	100
4.5 M H_2_SO_4_	50 °C	2
4.5 M H_2_SO_4_	Ambient	<1.5
4 M HCl	Ambient	<1.5
NTA	Ambient	<1.5 (*)
Sodium EDTA/HCl	Ambient	<1.5 (*)
EDTA acid/HCl	Ambient	<1.5 (*)

* Inconclusive results, solutions were not stable under the applied conditions.

**Table 2:** Sc/impurity metals concentration ratio in the initial FeCl_2_ solution and the three final 3.5 M HF Sc-rich eluate solutions.

Solution	Scenario	Sc/Ti	Sc/Zr	Sc/V	%Elution of Sc with 3.5 M HF
Initial Solution	FeCl_2_ solution	0.23	0.04	0.03	-
3.5.M HF elution	Two-step Elution Scrubbing with 0.1 M HF	3.58	3.13	29.14	94.62
	Two-step Elution Scrubbing with 0.5 M HF	4.57	4.40	131.25	78.02
	One-Step elution	1.33	0.25	3.86	99.58

**Table 3:** Concentration of initial and final streams of the IX process with the DEHPA-impregnated resin.

	Zr, ppm	V, ppm	Ti, ppm	Sc, ppm
FeCl_2_ solution	3680	4200	530	130
First step 0.1 M HF	194.00	8.50	23.50	<2.5
Second 3.5 M HF	156.03	28.05	89.00	305.00

## Data Availability

The original contributions presented in this study are included in the article. Further inquiries can be directed to the corresponding author.
